# Biosynthesis of Zinc Oxide Nanoparticles on l-Carnosine Biofunctionalized Polyacrylonitrile Nanofibers; a Biomimetic Wound Healing Material

**DOI:** 10.1021/acsabm.3c00499

**Published:** 2023-09-18

**Authors:** Shahin Homaeigohar, Mhd Adel Assad, Amir Hossein Azari, Farnaz Ghorbani, Chloe Rodgers, Matthew J. Dalby, Kai Zheng, Rongyao Xu, Mady Elbahri, Aldo. R. Boccaccini

**Affiliations:** † School of Science and Engineering, University of Dundee, Dundee DD1 4HN, U.K.; ‡ Nanochemistry and Nanoengineering, Department of Chemistry and Materials Science, School of Chemical Engineering, Aalto University, Espoo 02150, Finland; § Institute of Biomaterials, Department of Materials Science and Engineering, University of Erlangen-Nuremberg, Erlangen 91058, Germany; ∥ Centre for the Cellular Microenvironment, University of Glasgow, Glasgow 11 6EW, U.K.; ⊥ Jiangsu Province Engineering Research Center of Stomatological Translational Medicine, Nanjing Medical University, Nanjing 210029, China; # Department of Oral and Maxillofacial Surgery, Stomatological Hospital, Nanjing Medical University, Nanjing 210029, China

**Keywords:** nanofiber, biofunctionalization, wound healing, zinc oxide, polyacrylonitrile, l-carnosine

## Abstract

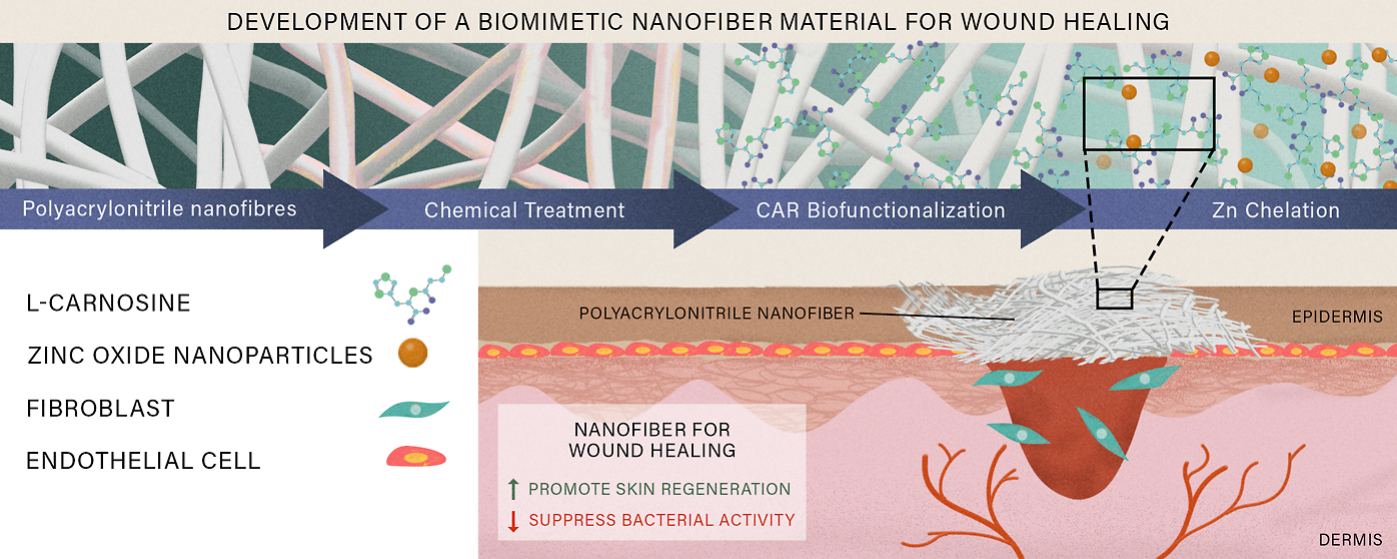

Multifunctional biohybrid nanofibers (NFs) that can simultaneously drive various cellular activities and confer antibacterial properties are considered desirable in producing advanced wound healing materials. In this study, a bionanohybrid formulation was processed as a NF wound dressing to stimulate the adhesion and proliferation of fibroblast and endothelial cells that play a major role in wound healing. Polyacrylonitrile (PAN) electrospun NFs were hydrolyzed using NaOH and biofunctionalized with l-carnosine (CAR), a dipeptide which could later biosynthesize zinc oxide (ZnO) nanoparticles (NPs) on the NFs surface. The morphological study verified that ZnO NPs are uniformly distributed on the surface of CAR/PAN NFs. Through EDX and XRD analysis, it was validated that the NPs are composed of ZnO and/or ZnO/Zn(OH)_2_. The presence of CAR and ZnO NPs brought about a superhydrophilicity effect and notably raised the elastic modulus and tensile strength of Zn-CAR/PAN NFs. While CAR ligands were shown to improve the viability of fibroblast (L929) and endothelial (HUVEC) cells, ZnO NPs lowered the positive impact of CAR, most likely due to their repulsive negative surface charge. A scratch assay verified that CAR/PAN NFs and Zn-CAR/PAN NFs aided HUVEC migration more than PAN NFs. Also, an antibacterial assay implied that CAR/PAN NFs and Zn-CAR/PAN NFs are significantly more effective in inhibiting *Staphylococcus aureus* (*S. aureus*) than neat PAN NFs are (1000 and 500%, respectively). Taken together, compared to the neat PAN NFs, CAR/PAN NFs with and without the biosynthesized ZnO NPs can support the cellular activities of relevance for wound healing and inactivate bacteria.

## 1 Introduction

Globally, acute or chronic wounds and, in general, skin irregularities endanger patients’ welfare and majorly challenge the healthcare systems.^1^ Almost 1–2% of the general population experience a chronic wound, and 25% of diabetic patients suffer from an ulcer.^2^ To properly circumvent this crisis, the global wound care market is forecast to exceed £18.6 billion by 2024 from £14.8 billion in 2019.^3^ This market is rapidly growing to meet the needs of a large population of patients through the provision of state-of-the-art technologies and products.^4^ In this regard, classic protective barriers have been modernized as interactive multifunctional wound dressings that encourage wound healing by stimulating the cell proliferation and migration and controlling the healing cascade.^4^ Such sophisticated goals are satisfied only by the improved design and fabrication of advanced, multipurposed wound healing (dressing) materials.

Commercial wound dressings are mainly in the form of foams, sponges, hydrogels, and films.^1^ As a newer type of wound dressing material, NFs have been proven to provide distinct structural advantages. NF meshes comprise overlapping NFs, where diameter ranges from a few hundred nanometers to a few microns. This unique structure provides an extremely large available surface area and mimics the extracellular matrix (ECM), thereby improving the interaction with the cells involved in the wound healing process.^5^ The small interstices and vast surface area of the NF wound dressings can also raise hemostasis. Having a small pore size and superb conformability, NF dressings can shield a wound against the invasion of bacteria (and resulting infection) and the ingrowth of cell/tissue. This feature can be regarded as an advantage for NF dressings over their microfibrous and mesh commercial counterparts.^6^ Most importantly, in comparison to commercial wound dressings, the large surface area of NF wound dressings offers a promising platform for loading of drugs and bioactive agents. As a result, not only does the NF structure biomimic the ECM physically but also allows the provision of sufficient biochemical cues to cells to provoke their regenerative activities. In this regard, in our recent studies, we developed bovine serum albumin (BSA)-functionalized polyacrylonitrile (PAN)^7^ and poly(ε- caprolactone) (PCL)^8^ NF wound dressings that could stimulate fibroblast and keratinocyte cells to enhance proliferation and thus wound healing activity. In the former study, the BSA ligands exposed on PAN NFs could form a calcium-deficient hydroxyapatite shell and further activate the cells because of the release of Ca^2+^ and phosphate ions into the cell culture medium.

In the present study, instead of using the large biomolecule of BSA that could readily fold/unfold with pH/temperature changes and form clusters that reduce the pore size and challenge air/water permeability and wound exudate uptake, l-carnosine (CAR) is loaded on the surface of PAN NFs. It is important to note that PAN was intentionally selected as a synthetic, non-degradable NF material. Compared to the natural polymers studied for wound healing applications, *e.g.*, chitosan, alginate, *etc.*, synthetic polymers show distinct advantages, including proper physicochemical properties, scalability, relatively low cost, easy processability, and the possibility of integration into industrial technologies.^9^ Such engineering characteristics are of paramount importance with respect to the translation and commercialization of the products made of these polymers. Additionally, PAN is a synthetic non-degradable polymer that compared to synthetic bio-degradable polymers, such as poly-l-lactic acid (PLA) and poly-l-glycolic acid (PLGA) that are largely used for the development of wound dressings, does not trigger an inflammatory and fibrotic response.^10^ PAN is also more electrospinnable than natural polymers, with no need to use hazardous catalysts and chemical cross-linkers, and is readily adapted to advanced electrospinning technology designed for scalable NF production.

While PAN NFs can effectively create a biomimetic physical structure, their surface decoration with proteins and peptides, as biochemical cues, can raise their biofunctionality and cell interactivity. In this regard, in the current study, CAR is immobilized on PAN NFs. CAR is an endogenous dipeptide (comprising β-alanine and l-histidine) commonly found in cells with long lifespans, such as muscle cells and nerve cells.^11^ It has been shown to offer a variety of biological activities, such as antiaging, metal ion chelating, pH buffering, antiglycation, and antioxidation activity.^12^ As a result, CAR can play a role in the treatment of various age-related diseases^12^ and even in the healing of surgical wounds.^13^ The latter therapeutic effect originates from its supportive role in the longevity of fibroblasts^14^ and in nitrogen oxide (NO) production by endothelial cells.^15^ On the other hand, CAR protects cells against reactive oxygen species (ROS) and oxidative stress, a common feature of the pathogenesis of chronic ulcers.^16^

The multifunctionality of CAR/PAN NFs is assumed to be extended by employing the metal-chelating activity of CAR. In this regard, Zn has been previously coupled with CAR to develop a Zn-CAR complex, polaprezinc (PLZ), with various therapeutic potentials for wound healing.^11^ Therapeutic ions, including Mg, Ca, and Si, notably contribute to tissue repair and the regulation of cell metabolism.^17,18^ Additionally, Zn is an essential trace element taking part in the chemistry of different transcription factors or enzymes that contribute to cell proliferation, protein synthesis, and healing upon injury.^19^ Additionally, due to its immunomodulatory activity,^20^ Zn majorly contributes to wound healing. Zn is known as a micronutrient that contributes to the wound healing process through involvement in coagulation, membrane repair, angiogenesis, oxidative stress modulation, inflammation and immune defence, tissue re-epithelialization, and fibrosis/scar formation.^21^ Given the loss of Zn during injury, Zn supplementation has been shown to provoke wound healing in zinc-deficient patients.^19^ Thanks to its antioxidant activity, topical ZnSO_4_ (3%) has been extensively applied for wound healing.^22^ The other Zn-based compounds that are used for wound healing include ZnCl_2_ (1%) and ZnO. ZnO enables a prolonged supply of Zn to assure a higher wound healing efficiency.^21^ Moreover, ZnO intensifies collagen degradation within the necrotic wounds.^23^ It is worth noting that ZnO NPs have developed focus for their potential as a drug delivery carrier for wound healing, thanks to their effective cell penetration, immunomodulation, and antimicrobial activity.^24^

PLZ is a complex made of CAR and Zn (1:1 molar ratio) with a wound-healing effect that arises from the synergy between the two components.^11^ As shown through an *in vivo* study, PLZ can expedite the healing process of skin incisions.^25^ Sakae *et al.*(11) validated that PLZ and CAR can equally accelerate the healing of pressure ulcers after 4 weeks. PLZ is largely employed as a drug for the treatment of gastric ulcers in Japan, and in Europe and the US, both PLZ and CAR are available in the market as dietary supplements (with usual daily dosages of 75 mg and 130–600 mg for PLZ and CAR, respectively).^11^ In this study, we aim to create a NF wound dressing material based on PLZ-like compound (Zn-CAR) loaded PAN NFs that is assumed to promote wound healing by increasing the proliferation of fibroblasts and endothelial cells, thanks to the supportive role of Zn and CAR.

## 2 Experimental Section

### 2.1 Materials

PAN (Mw = 150,000), *N, N*-dimethylformamide (DMF), sodium hydroxide, zinc chloride, and l-carnosine were all obtained from Sigma-Aldrich (Darmstadt, Germany). Phosphate buffered saline (PBS) was purchased from Carl Roth GmbH (Karlsruhe, Germany). All the materials were used as received.

### 2.2 Sample Preparation and Functionalization

Several 20 × 20 cm^2^ PAN NF mats were prepared through electrospinning. Briefly, an 8 wt % (w/w) PAN/DMF solution was fed continuously into a syringe at the feed rate of 0.8 mL/h using a syringe pump (Harvard Apparatus, USA). The PAN NFs were collected on an aluminum foil situated 25 cm far from the nozzle with an applied voltage of 15 kV (Heinzinger Electronic GmbH, Germany) at room temperature and at the relative humidity of 70–75%. The collected NF mats were eventually dried overnight in a vacuum oven at 100 °C to remove any residual solvent.

**Figure 1:**
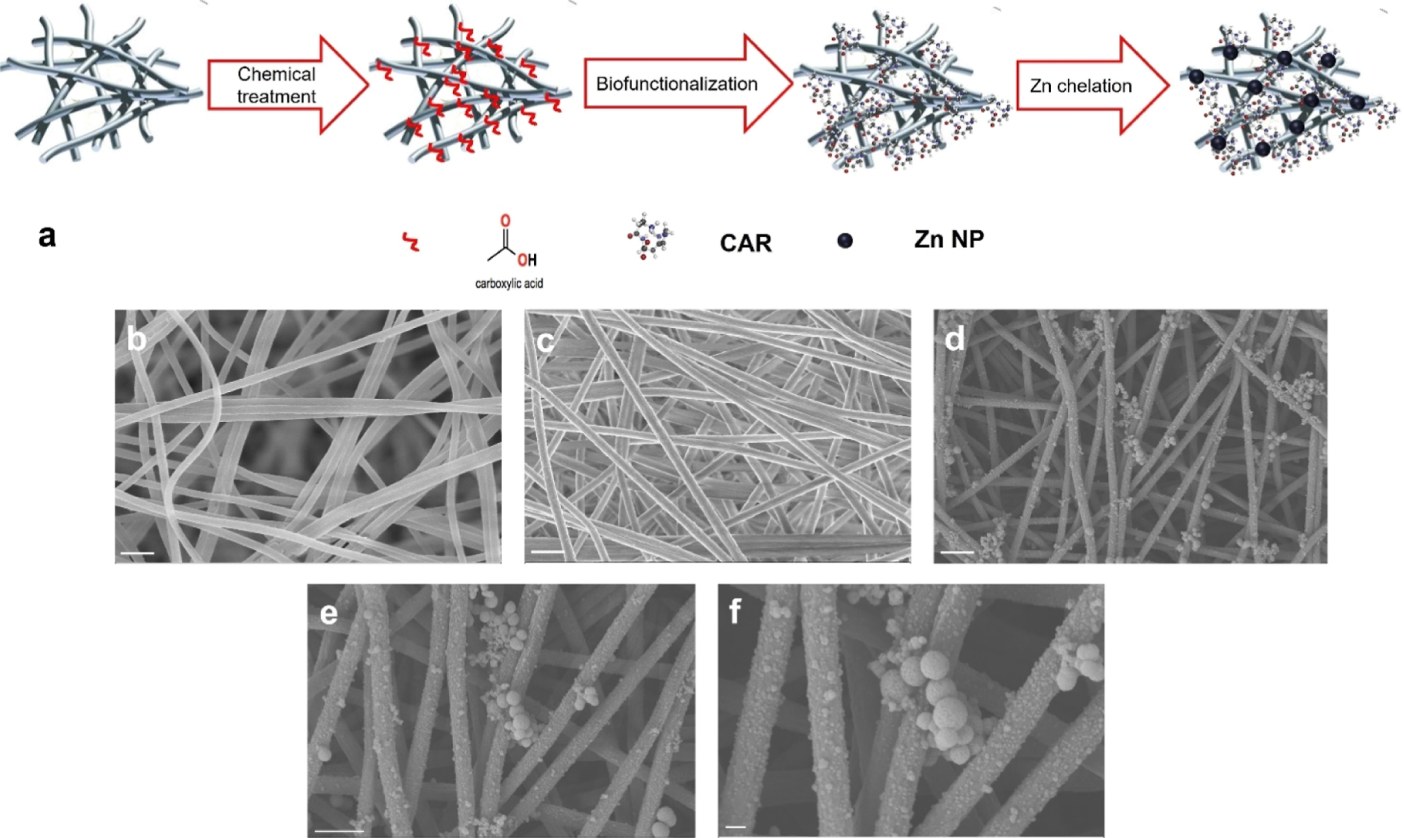
(a) Schematic illustration of the preparation process of Zn-CAR/PAN NFs through chemical treatment (hydrolysis), biofunctionalization, and Zn chelation, respectively. SEM images of the neat PAN (b, scale bar: 1 μm), CAR/PAN (c, scale bar: 1 μm), and Zn-CAR/PAN NFs (d–f, scale bars represent 1 μm, 1 μm, and 200 nm, respectively). Majority of the ZnO NPs are homogeneously distributed across the NF surface.

The PAN NF mats were first hydrolyzed in a 1 N NaOH solution for 2 h at 60 °C under continuous shaking. The hydrolyzed NFs were then washed thrice with distilled water and then air dried. This process leads to the emergence of hydroxyl and carboxyl groups on the NF’s surface. The hydrolyzed NFs were subsequently soaked into a CAR/PBS (5 mg/mL) solution for 5 h at 50 °C under continuous shaking. The biofunctionalized NFs were washed thrice with PBS and eventually air dried. The presence of carboxyl/hydroxyl groups on the NFs surface and their physicochemical bonding with the amine/carboxyl groups of CAR can assure proper biofunctionalization of the PAN NFs. For instance, esterification between the hydroxyl groups of the hydrolyzed PAN NFs and the carboxyl groups of CAR can result in the stable attachment of CAR on the NFs. The biofunctionalized NFs were later loaded with ZnO nanoparticles (NPs) *via* immersion in a ZnCl_2_ aqueous solution (20 wt %) for 2 h at room temperature. The resulting NFs were eventually washed thrice with deionized water to remove the remaining Cl and air dried. The entire procedure of functionalization of PAN NFs and their subsequent Zn loading is illustrated in Figure 1a.

### 2.3 Morphological Analysis

The morphology of neat PAN, CAR/PAN, and Zn-CAR/PAN NFs was characterized by scanning electron microscopy (SEM) (Zeiss Sigma VP Gemini from Carl ZEISS, Germany) after sputter coating of the NFs with Au/Pd NPs (5 nm). Based on SEM images and measuring the diameter of 50 randomly selected NFs by the ImageJ software, a diameter histogram of all classes of the NFs was constructed.

### 2.4 Elemental Analysis

The elemental composition of the NFs was identified by using energy dispersive spectroscopy (Oxford Instruments, Abingdon, UK) under a 20 kV applied voltage.

### 2.5 Wound Exudate Capacity

The wound exudate uptake capacity of the NF mats was determined based on their weight gain at the pre-determined time intervals of 1, 3, and 7 h when submerged in an exudate mimicking solution at 37 °C. This solution was previously prepared by dissolving 0.37 g of calcium chloride (Sigma-Aldrich, USA) and 8.3 g of sodium chloride (Sigma-Aldrich, USA) in 1 L of water according to a standard protocol.^26^ The exudate uptake capacity of the NF mats was quantified through the following eq 1
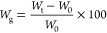
1where *W*_g_, *W*_0_, and *W*_t_ represent weight gain (*i.e.*, uptake capacity) and the initial and momentary weight of the NF mats before and after immersion in the solution.

### 2.6 Mechanical Testing

The tensile elastic modulus and tensile strength of the NF samples (3 cm × 1 cm × 0.1 cm) were measured by a uniaxial tensile tester (Universal Tester Instron 4204, 1000 N load cell). From each group, five samples were tested.

### 2.7 Porosity and Pore Size Measurement

The porosity of the NF samples was quantified based on the weight and volume of three cuboid NF samples (with given dimensions). To do so, first the apparent density (ρ_0_) of the NF samples was calculated from their volume and mass. Subsequently, porosity (ε) was determined *via*eq 2(27)
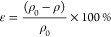
2where ρ is the bulk density of PAN (1.184 g/cm^3^^28^). Having calculated the porosity [ε(−)] and NF’s diameter [*d* (nm)], the mean pore radius () of the NF mats can be measured through the following equation^29^
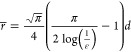
3

### 2.8 Surface Chemical Analysis

Surface chemical analysis of the NFs was carried out by ATR-FTIR spectroscopy (ALPHA (ATR-Ge, ATR-Di) from BRUKER Optik GmbH, Ettlingen, Germany).

### 2.9 Crystalline Structure Analysis

The crystalline structure of the NF samples was characterized at ambient temperature *via* X-ray diffractometry (Miniflex 600, Rigaku, Japan) with Cu-Kα radiation (λ = 0.15418 nm).

### 2.10 Water Contact Angle Measurement

Hydrophilicity of the NF mats was quantified through water contact angle measurements (DSA 30, Krüss, Germany) and by mounting a 3 μL water droplet on several spots of the NF mats.

### 2.11 Water Vapor Permeability Measurement

The water vapor permeability of the NF mats was determined following the ASTM E96 standard. To do so, the NF mats were fixed on the mouth (176 × 10^–6^ m^2^) of several 5 mL storage vials with screw caps. These vials were previously filled with distilled water (4 mL). The water-containing vials sealed with a NF mat were placed within a shaking incubator at 37 °C for 72 h. Afterward, the vials were weighed, and the water vapor transmission rate (WVTR) was measured using eq 4(30)
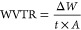
4where Δ*W* indicates the water weight loss (g), *t* is the interval at which the weight loss is measured (h), and *A* represents the permeation area (m^2^).

### 2.12 L929 and HUVEC Cell Viability

Cell viability in proximity of the neat PAN, CAR/PAN, and Zn-CAR/PAN NFs was characterized using a CCK8 cell proliferation kit (APExBIO, Cat# K1018). The NF samples were cultured with HUVECs or L929 cells at the density of 5 × 10^3^ cells/well within a 96-well plate for 1 and 4 days. Before each measurement, 10 μL of the CCK8 agent was added into the wells in the dark, and the plates were incubated at 37 °C for 1.5 h. The optical absorbance was subsequently measured at the wavelength of 450 nm using an ELISA plate reader (Biotek, Winooski, VT, USA).

### 2.13 Live/Dead Assay

To visualize the live/dead cells, the NF samples were cultured with HUVECs or L929 cells (5 × 10^3^ cells/well) within a 12-well plate for 24 h. The cells were stained with Calcein-AM and PI for 30 min using a live/dead assay kit (Cat# C2015S, Beyotime, Shanghai, China) and imaged by a confocal fluorescence microscope. The Calcein-AM^+^ cells (green) were considered as live cells, while the dead cells featuring damaged plasma membranes were marked with red fluorescence.

### 2.14 DAPI/Phalloidin Staining Assay

To monitor the cell morphology (cytoskeleton), the cells were fixed with 4% paraformaldehyde for 30 min. After washing three times with PBS, the cells were blocked by 5% BSA for 1 h at 37 °C. Subsequently, the cells were incubated with Actin-Tracker Green-488 (1:100; Cat# C2201S, Beyotime, Shanghai, China) at room temperature for 1 h in a dark environment. The Actin-Tracker Green-488 probe is a phalloidin labeled by the fluorescent dye Alexandra Fluor 488. The nuclei were counterstained with a DAPI solution (Cat# C1005, Beyotime, Shanghai, China). The stained cells were imaged by using a confocal fluorescence microscope.

### 2.15 Scratch Wound Healing Assay

Cell migration driven by the presence of the NF samples was monitored through a scratch wound healing assay. To do this, the NF mats (6 cm^2^ in area) were immersed into 1 mL of DMEM at 37 °C for 24 h. The leach liquor was then collected to incubate HUVECs. Subsequently, a 100 μL pipette tip was used to introduce a scratch wound in the center of the HUVEC monolayer. After washing with PBS, the cells were cultured for 20 h in a serum-free medium, and the scratch area was imaged. The cell migration ratio was quantified using ImageJ software.

### 2.16 Antibacterial Testing

The antibacterial activity of the NF mats against *Staphylococcus aureus* (*S. aureus*) bacteria was tested through the Oxford cup method. Briefly, a *S. aureus* bacteria suspension was diluted down to the concentration of 10^5^–10^6^ cfu/mL. Afterward, a 0.1 mL aliquot of the bacterial suspension was evenly spread on the surface of LB agar. The center of the Oxford cup (Φ 7.8 mm) was placed on the surface of the medium symmetrically, and 100 μL of leach liquor of the three NFs was injected into each Oxford cup. The plate was incubated at 37 °C for 24 h, and the diameter of the inhibition zone was observed and measured. Additionally, the antibacterial efficiency of the NFs was monitored through a live/dead staining assay. To do this, the logarithmic growth stage of *S. aureus* was considered, and the bacterial concentration was diluted down to 10^5^–10^6^ cfu/mL with tryptone soy broth. The leach liquor of the NFs was added to the bacteria suspension based on the medium: leach liquor ratio of 1:10 (v/v), and the entire medium was incubated at 37 °C for 24 h. Afterward, the bacteria were stained using the SYTO 9/propidium iodide (PI) Live/Dead Bacterial Double Stain Kit (Maokangbio, Shanghai, China). For this purpose, equal amounts of SYTO 9 and PI were mixed within a microcentrifuge tube and were then added to the bacteria suspension according to a fixed ratio of 3 μL of dye per 1 mL of the suspension. The mixture was incubated at room temperature in the dark for 15 min. After staining, 5 μL of stained bacterial suspension was aspirated into a slide (covered with an 18 mm^2^ coverslip) and observed using a fluorescence microscope.

### 2.17 Statistical Analysis

The biological data were statistically analyzed *via* one-way analysis of variance (ANOVA). The *p*-values less than 0.05 implied a significant difference between the compared biological data.

## 3 Results and Discussion

### 3.1 Morphology of Biosynthesized ZnO NPs and Bio(nano)hybrid NFs

The camera image shown in Figure S1 demonstrates the physical appearance of a Zn-CAR/PAN NF mat. The morphology of neat PAN NFs, CAR/PAN NFs, and Zn-CAR/PAN NFs is shown in Figure 1b–f. While the surfaces of neat PAN NFs and CAR/PAN NFs are similar in morphology, the surfaces of Zn-CAR/PAN NFs are fully covered with small ZnO NPs. The majority of ZnO NPs are homogeneously distributed across the CAR/PAN NFs, implying the successful chelation and formation of Zn-rich zones along the NFs thanks to proper biofunctionalization and homogeneous distribution of the CAR ligands within the nanostructure. A similar behavior was seen in an earlier study of ours that dealt with the formation of TiO_2_ NPs on the surface of polyether sulfone (PES) NFs through a sol–gel process.^31^ This comparable outcome shows the efficiency of the biofunctionalization approach in the synthesis of ultrafine, well-distributed ZnO NPs on the NF surface. The PAN NFs were bead-less and their average diameter was 305 ± 55 nm. Compared to the neat NFs, the diameter of CAR/PAN NFs does not grow significantly and was measured to be ∼300 ± 40 nm. On the other hand, upon formation of ZnO NPs, the NF diameter rose to 405 ± 45 nm. Figure S2a−c shows a diameter histogram for the three groups of NFs. It is worthy to note that the average diameter of Zn-CAR/PAN NFs matches well the diameter range of collagen NFs in skin’s ECM (*i.e.*, 50–500 nm^32^), thus assuring a proper biomimicry effect and cell-material interaction.

In a parallel study, a second group of Zn-CAR/PAN NFs was prepared without thorough washing after immersion of CAR/PAN NFs into a ZnCl_2_ aqueous solution. As seen in Figure S3a,b, the surface morphology of the resulting NFs is totally different than that of the properly washed Zn-CAR/PAN NFs. This distinct surface phase could originate from the remaining Cl and be partially composed of ZnCl_2_, as verified *via* an EDX analysis (Figure S3c–f).

### 3.2 Porous Structure of the Bio(nano)hybrid NFs

**Figure 2:**
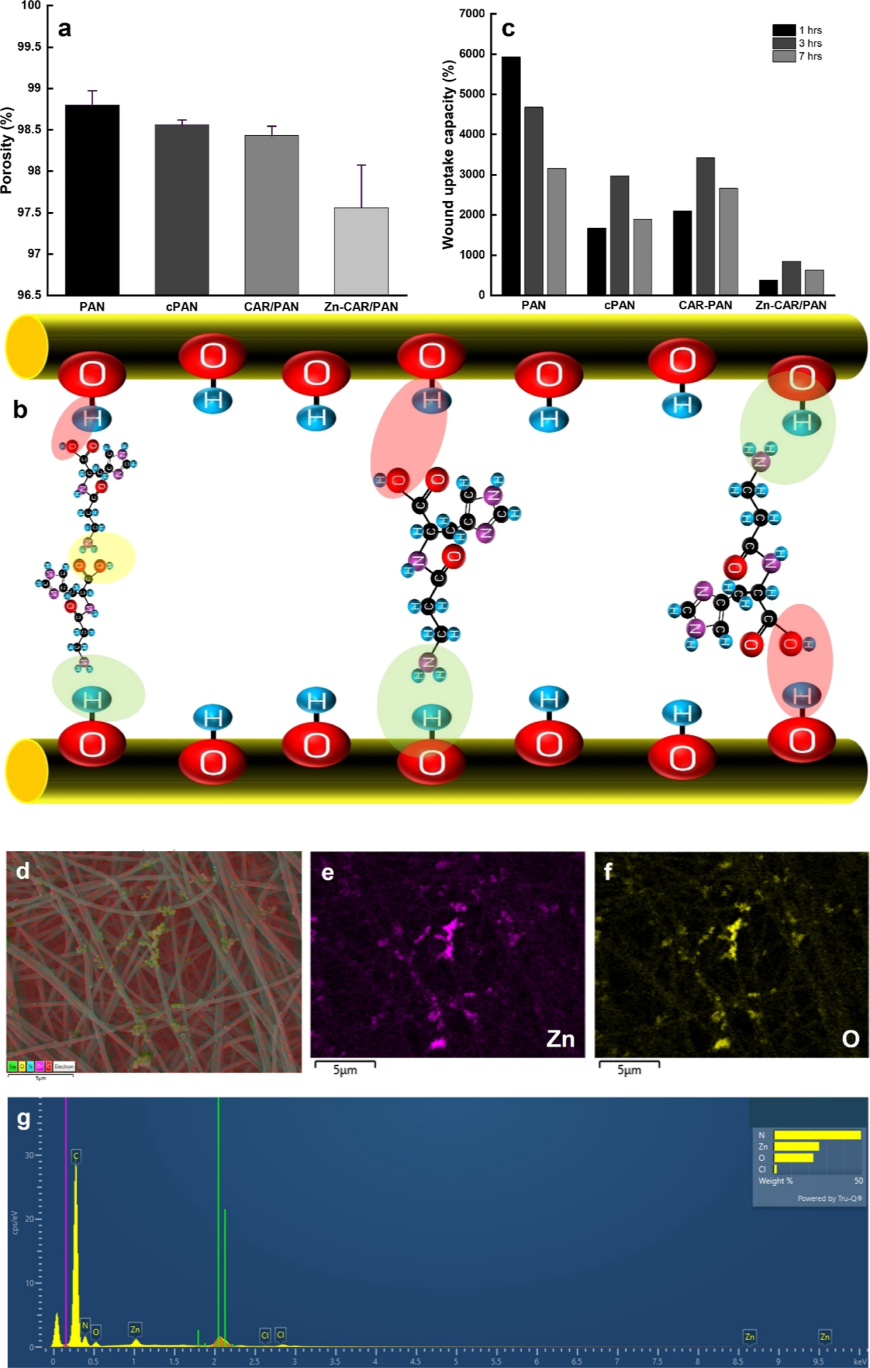
(a) Porosity of CAR/PAN NF mats with or without ZnO NPs compared to those of the neat and hydrolyzed PAN NF mats. (b) Schematic illustration of inter-fiber cross-lining mediated by CAR ligands through hydrogen bonding (green zone), esterification (red zone), and peptide bonding (yellow zone) between the hydrolyzed PAN NFs and CAR. (c) Wound exudate uptake capacity of CAR/PAN NF mats with or without ZnO NPs compared to those of the neat and hydrolyzed PAN NF mats. EDX analysis verifies the formation of Zn-rich zones along the NFs: (d) general elemental map (the elements have been represented by colorful dots. N might indicate the amine groups of CAR), (e) corresponding Zn map of (d), (f) corresponding O map of (d), and (g) elemental analysis of a selected area of Zn-CAR/PAN NF mat.

Porosity of NF mats is crucial in terms of cell-material interactions, exudate uptake capacity, and water vapor permeability. The NF diameter distribution and the presence of secondary materials (*e.g.*, ZnO NPs and CAR) on the NFs can potentially affect the porosity and pore size of the NF mats. As calculated *via*eq 2 and shown in Figure 2a, the porosity of the PAN NF mats does not decline notably after hydrolysis, CAR biofunctionalization, and surface decoration with ZnO NPs and varies within a limited range of 98.8–97.6% from the neat PAN NF mat to the Zn-CAR/PAN NF mat. This porosity deals with the bulk of the NF mats and can be considered as a 3D porosity. The porosity that the cells will encounter, though, will be at the outermost NF surface layers and is a quasi-2D porosity. To roughly determine this porosity, SEM images of the neat, CAR/PAN, and Zn-CAR/PAN NF mats were analyzed using ImageJ. According to Figure S4a−c, the surface porosity can range from 17% for the neat PAN NF mat down to 6 and 8% for CAR/PAN and Zn-CAR/PAN NF mats, respectively. It is assumed that interfiber cross-linking induced by the presence of CAR ligands could lower the porosity. Not only can the immobilized CAR ligands bond to each other through peptide bonds but also to another hydrolyzed PAN NF through esterification or hydrogen bonding (Figure 2b). This behavior was similarly reported by Elbahri *et al.*(33) for a BSA/poly(acrylonitrile-*co*-glycidyl methacrylate) (PANGMA) NF membrane, whose surface porosity and pore size declined due to BSA-induced interfiber cross-linking, resulting in an optimized size selectivity to coarse water pollutants. However, by getting involved in the chelation of Zn^2+^ ions and forming ZnO NPs, some parts of the CAR ligands are consumed, and thus the cross-linking intensity drops. This consequence can potentially lead to a slightly higher surface porosity for Zn-CAR/PAN NFs. The pore size of the NF mats, measured through eq 3 with inclusion of the approximate bulk porosity values above mentioned, was 39.4, 29.5, and 26.35 μm for neat PAN, CAR/PAN, and Zn-CAR/PAN NF mats, respectively. On the other hand, in case, the surface (2D) porosity values are included in eq 3, the pore size can be measured to be 138 nm (neat PAN NF mat), 38 nm (CAR/PAN NF mat), and 332 nm (Zn-CAR/PAN NF mat). Surprisingly, the pore size of the Zn-CAR/PAN NF mat is ∼140% larger than that of the neat PAN NF mat, most likely due to the increased negative electrostatic charge of the NFs, arising from a large number of the oxygen-bearing functional groups that emerged after hydrolysis and formation of the ZnO NPs. Knowing that fibroblasts are typically as large as 10–15 μm,^34^ and the common bacteria involved in wound infection are as large as 0.5–3 μm [*Escherichia coli* (*E. coli*): 1–2 μm,^35^ and *S. aureus*: 0.5–1 μm,^36^ it is assumed that the NF mats would resist against the penetration of both cells and bacteria into them. Therefore, the NF mats properly act as a physical barrier against bacteria invasion into the wound and cell/tissue ingrowth. The latter effect matters during dressing replacement, as low cell adhesion minimizes wound pain and the destruction of the newly formed granulation tissue.

### 3.3 Wound Exudate Uptake Capacity and Hydrophilicity of the Bio(nano)hybrid NFs

The porosity of a NF wound dressing dictates its structural performance, such as wound exudate uptake capacity and water vapor permeation rate. A suitable wound dressing is permeable to water vapor and maintains a moist exudate without pooling. The wound dressing should also prevent extensive liquid absorption and evaporation, likely leading to desiccation of the wound bed. Figure 2c shows the wound exudate uptake capacity of the NF dressings with or without CAR and ZnO NPs. As seen in this figure, the neat PAN NF mat has a larger exudate capacity than the hydrolyzed PAN, CAR/PAN, and Zn-CAR/PAN NF mats. Thanks to the higher polarity of CAR/PAN NFs with or without ZnO NPs compared to the neat PAN NFs, the hydrated biofunctionalized NF mats with hydrophilic CAR ligands and ZnO NPs are as swollen and thus lose their specific surface area and porosity. As a result, a lower wound exudate uptake capacity is recorded for these groups of NF mats. A similar behavior was observed in our earlier study on BSA/PCL NF dressings, where hydration-induced swelling of BSA could challenge exudate absorption.^8^ The neat PAN NF mat reaches the saturation level after 1 h and then pumps out the absorbed exudate over time up to 7 h (Figure 2c). The exudate penetrates the PAN NF mat under a capillary force which originates from the Laplace pressure. The Laplace pressure (*P*_L_) can be calculated *via* the Young–Laplace equation (eq 5)^37^
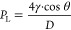
5where γ, θ, and *D* represent the surface tension of the exudate mimicking solution, the static contact angle of the NF mat, and the capillary diameter. According to eq 5, the capillary force and the Laplace pressure increase with a smaller pore size and a higher contact angle (hydrophobicity). Considering the hydrophobicity of the PAN NF mat and its small pore size (following that of CAR/PAN NFs), a large capillary force is responsible for the prompt absorption of exudate into the mat. However, as mentioned earlier, after saturation for 1 h, exudate seems to be pumped outward. This performance might arise from the gradual swelling of PAN NFs due to the penetration of metal ions and/or water molecules into the intermolecular spacing of the polymer. Since a favorable wound dressing should be able to provide solely a unidirectional water-transport effect to pump out the wound exudate and prevent it from reverse osmosis, the PAN NF mat’s ideal performance lasts only 1 h. Differently, the hydrolyzed PAN, CAR/PAN, and Zn-CAR/PAN NFs hold a lower amount of exudate compared to the PAN NFs, are saturated after 3 h, and pour out the exudate thereafter. These classes of NFs are (super) hydrophilic, as verified through a water contact angle measurement test. While PAN NFs show a water contact angle of 140°, the hydrolyzed PAN NFs, CAR/PAN NFs, and Zn-CAR/PAN NFs show the contact angle of almost 0° (Figure S5a−d). As a result of hydrophilicity, the capillary force declines, and thus a lower amount of exudate over a longer time is absorbed into the NF mats. On the other hand, the hydrophilic nature of such NFs turns out to be partially hydrophobic when contacting a plethora of metal ions that can cap the surface functional groups. As a result, the NF mats start to lose their exudate holding capacity and pump out some part of the exudate after 3 h. Therefore, these classes of NF mats keep a less amount of exudate but lose their effective holding capacity after a longer time, compared to the neat PAN NFs do.

### 3.4 Water Vapor Permeability of the Bio(nano)hybrid NFs

The WVTR for the neat PAN, hydrolyzed PAN, CAR/PAN, and Zn-CAR/PAN NF mat was measured to be 81, 81.23, 64.64, and 75 g·h^–1^·m^–2^, respectively. Despite a comparable bulk porosity, the biohybrid (CAR-containing) NF mats are less permeable than the neat and hydrolyzed PAN NF mats, implying that surface chemistry and the presence of CAR on the NF’s surface play a role in their water vapor permeation behavior. Presumably, water is condensed on the NF surface and swells the CAR ligands. This hydration-induced swelling shrinks the pore size and reduces the porosity of the mat, thereby challenging the permeation of water vapor through the NF structure. Such behavior was already reported in our earlier study on the biohybrid NF mats composed of BSA and PCL.^8^ Moreover, interfiber cross-linking caused by the CAR ligands can exacerbate the water vapor permeation behavior of the biohybrid NF mat. The situation is less severe for Zn-CAR/PAN NF mat, as some functional groups of the CAR ligands are already occupied by ZnO NPs, and thus only a fraction of the CAR ligands are swollen by condensed water molecules.

### 3.5 Elemental Composition of the Bio(nano)hybrid NFs

The surface composition of the NF mats was characterized *via* EDX analysis. As shown in Figure 2d,e, the Zn-rich zones (NPs) are evidently spread across the NF mat. This is more obvious in the distinct spherical nanostructures located on the NF’s surface, which are assumed to be large clusters of ZnO NPs. Figure 2f highlights the presence of oxygen throughout the NF mat. There is a meaningful overlap of the Zn and O representing zones, implying the availability of ZnO NPs. Figure 2g demonstrates the distribution of the elements in the Zn-CAR/PAN NF mat in a quantitative manner. After C and N, which originate from either PAN or CAR, Zn is the third most abundant element. This implies that the biohybrid NFs can effectively reduce Zn^2+^ ions and form ZnO NPs.

### 3.6 Surface Chemistry of the Bio(nano)hybrid NFs

**Figure 3:**
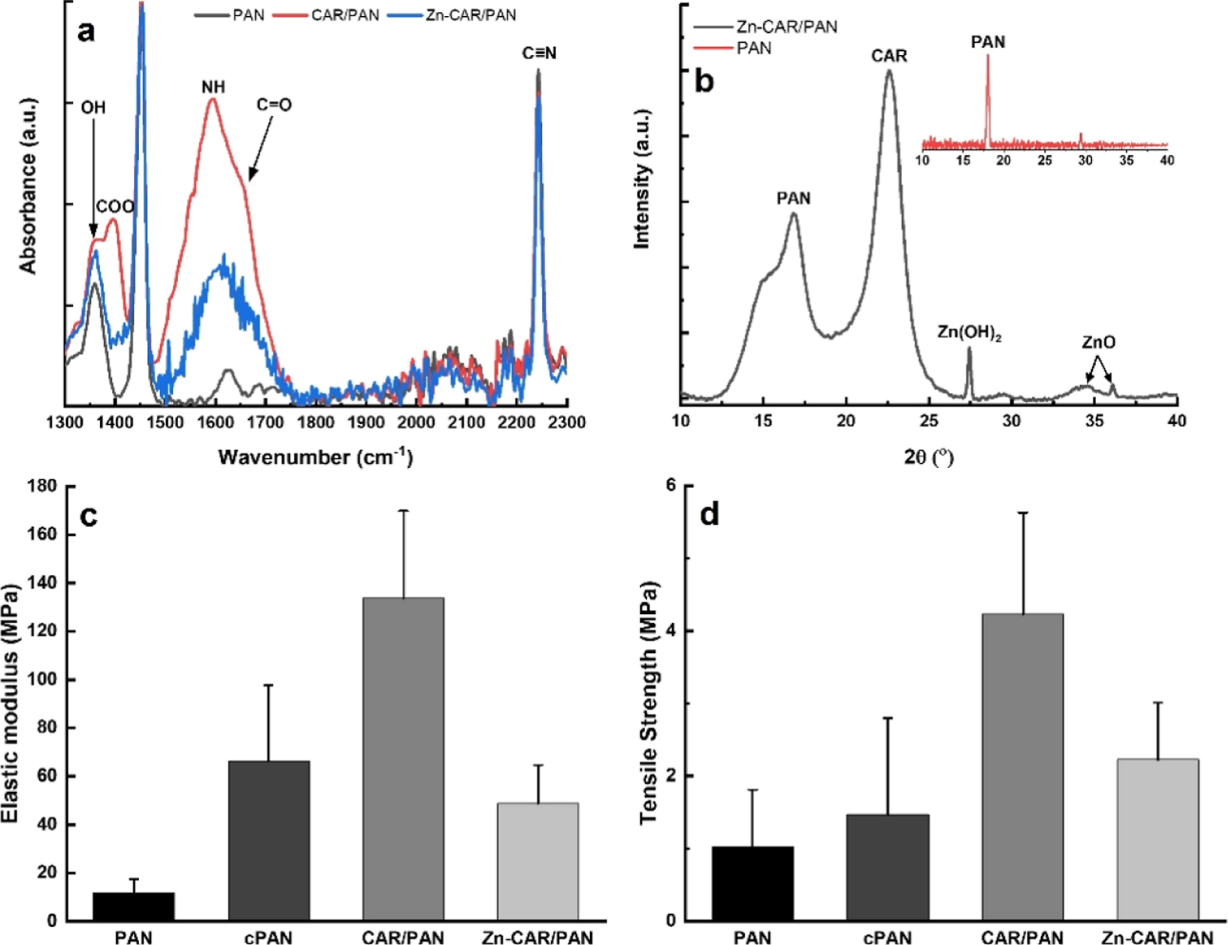
(a) ATR-FTIR spectra of neat PAN, CAR/PAN, and Zn-CAR/PAN NFs, (b) XRD spectra of Zn-CAR/PAN NFs (inset image shows the characteristic peak of PAN NFs), (c) elastic modulus, and (d) tensile strength of neat PAN, hydrolyzed PAN (cPAN), CAR/PAN, and Zn-CAR/PAN NF mats. The intra- and inter-fiber cross-linking of PAN NFs driven by the interaction of hydrolyzed PAN’s and CAR’s functional groups play a major role in the enhancement of the elastic modulus (stiffness) and tensile strength of the NF mats.

The successful biofunctionalization of PAN NFs with CAR and later Zn chelation by the CAR ligands were tracked *via* ATR-FTIR. Figure 3a shows the FTIR spectra for the neat PAN, CAR/PAN, and Zn-CAR/PAN NF mats. The characteristic peak seen at 2243 cm^–1^ represents CN stretching of PAN NFs. Evidently, the intensity of this peak declines for CAR/PAN and Zn-CAR/PAN NFs. On the other hand, in the case of the hydrolyzed PAN NFs, two strong characteristic peaks appear at 1355 cm^–1^ (OH bending) and 1680 cm^–1^ (C=O stretching) that indicate the successful hydrolysis of PAN NFs.^7^ These peaks shift for CAR/PAN NFs to 1360 and 1656 cm^–1^, implying hydrogen bonding between the amine groups of CAR and hydroxyl/carboxyl groups of the hydrolyzed PAN NFs. Additionally, two new strong characteristic peaks appear at 1397 cm^–1^ (COO^–^) and 1594 cm^–1^ (NH_3_^+^) that clearly represent the presence of CAR ligands on the PAN NFs.^38^ Interestingly, after immersion of CAR/PAN NFs into a ZnCl_2_ aqueous solution, the carboxyl band of CAR disappears, and the amide band shifts to 1611 cm^–1^ and its intensity notably declines. All these changes verify that Zn has been successfully chelated by CAR’s functional groups. In this regard, Wagner and Baran^38^ state that Zn^2+^ cations can be effectively chelated by CAR’s terminal amino N-atom and/or deprotonated amide N-atom and one carboxylate oxygen of CAR. As a multidentate ligand, CAR can enable the chelation of metal cations through six potential pathways/sites: one oxygen and nitrogen of the amide bond (O and N), one amino nitrogen (N), two imidazole nitrogen (N1 and N3), and one carboxylate oxygen (O). In the Zn-CAR complex, Zn^2+^ ions bind to the amino N, carboxyl O, and two N atoms of the imidazole group of CAR in the form of 4 coordination.^39,40^

### 3.7 Crystallinity of the Biosynthesized ZnO NPs

To further prove the existence of ZnO NPs on the NFs surface and to determine their crystallinity, the NFs were analyzed by XRD. Figure 3b shows several XRD peaks that can be attributed to the different components of the Zn-CAR/PAN NF system. For instance, the broad peak seen at 2θ = 17°, represents the (100) crystalline planes of PAN.^41,42^ Compared to the characteristic peak of the neat PAN located at 2θ = 18° (the inset image), this peak has slightly shifted and weakened (PAN has been further amorphized) due to chemical treatment (hydrolysis) and bonding with CAR. The peaks seen at 2θ = 23 and 27° can be attributed to CAR^43^ and Zn(OH)_2_,^44^ respectively. The low intensity peaks seen at 2θ = 34 and 36° represent ZnO (002) polycrystalline hexagonal Wurtzite structure^45^ and ZnO (101),^46^ respectively. According to these XRD peaks, it can be concluded that the NPs are made of either ZnO or Zn(OH)_2_ or Zn(OH)_2_/ZnO as a core–shell NP. The latter class of NPs might form due to the adsorption of humidity on the surface of ZnO NPs. Similarly, Zhou *et al.*(47) have reported the formation of a thin layer of Zn(OH)_2_ on ZnO quantum dots. To ascertain that the NPs are made of ZnO, the FTIR spectrum was double checked. The characteristic peak of Zn–O should have appeared at 618 cm^–1^,^48^ which overlapped with CAR’s carboxyl peak located at 627 cm^–1^,^38^ thus remained hidden. The crystallite size (*D*, nm) of ZnO and Zn(OH)_2_ [or Zn(OH)_2_/ZnO] NPs can be calculated through the Debye–Scherrer equation (eq 6)^49^
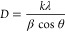
6where *k* = 0.94 (for spherical NPs), λ is the wavelength of X-ray radiation (0.1541 nm), and β is the full width at half-maximum (FWHM). According to this formula, the crystallite size of ZnO and Zn(OH)_2_ [or Zn(OH)_2_/ZnO] NPs is as small as ∼0.1 nm (1 Å) and 4 nm, respectively.

### 3.8 Mechanical Properties of the Bio(nano)hybrid NFs

The physicochemical interaction of CAR ligands with PAN, ZnO NPs, and even themselves is believed to lead to a more robust, stiff NF structure. Figure 3c,d shows the elastic modulus and tensile strength of the neat PAN, hydrolyzed PAN, CAR/PAN, and Zn-CAR/PAN NF mats extracted from their corresponding stress–strain curves (Figure S6). The PAN NF mat becomes much stiffer and stronger after the chemical treatment (hydrolysis), most likely due to intermolecular bonding between the PAN chains functionalized with oxygen-bearing functional groups. This effect is further intensified after the biofunctionalization of PAN NFs with CAR. The CAR ligands are thought to act as the cross-linkers connecting the NFs, thereby raising the stiffness and strength of the mat comprising thereof. A similar behavior has been previously reported for BSA-functionalized PAN NFs^7^ and BSA-functionalized PANGMA NFs,^33^ where biological nanowires interconnect the NFs and increase the netting points of the mat, thereby stiffening/strengthening the structure. As a result, inter/intrafiber bonding brings about a considerably higher elastic modulus/tensile strength as 462%/44% (cPAN), 1036%/315% (CAR/PAN), and 314%/118% (Zn-CAR/PAN) increase when comparing with that of the neat PAN NFs. The less significant increment of elastic modulus/tensile strength for Zn-CAR/PAN NFs can be justified by the occupation of a noticeable fraction of CAR’s functional groups with ZnO NPs and thus the reduced contribution of CAR to the interfiber cross-linking process. An ideal wound dressing needs to be sufficiently pliable and elastic and yet mechanically strong to prevent excessive damage to the wounded tissue.^50^ The mechanical stiffness of a wound dressing material impacts on cellular activities, as cell-material interactions depend on the applied shear stresses and mechanical signaling channels that govern the cell migration, proliferation, and differentiation.^51^ Optimally, the mechanical properties of a wound dressing material must be in harmony with those of the skin tissue being treated to offer similar biomechanical signals. Depending on the origin of the skin, the elastic modulus of human skin lies in the range of 8 kPa up to 70 MPa.^52^ The elastic modulus of Zn-CAR/PAN NF dressing is 48.7 ± 16 MPa, that optimally matches this range.

### 3.9 L929 Fibroblast Cell Viability and Adhesion on the Bio(nano)hybrid NFs

**Figure 4:**
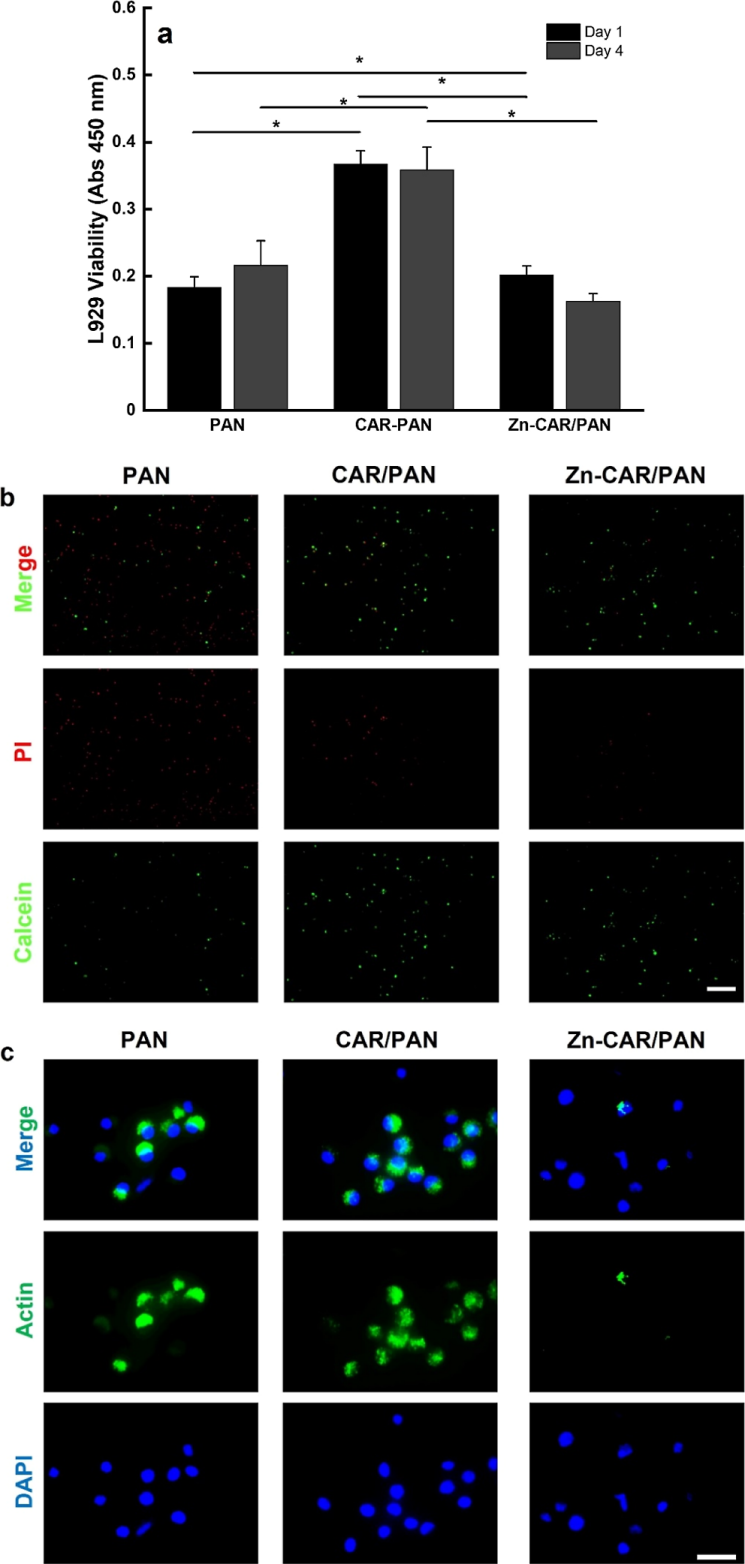
(a) L929 cell viability in the proximity of neat PAN, CAR/PAN, and Zn-CAR/PAN NFs (*: *p* < 0.05). On day 1, the cell viability of PAN NFs is significantly inferior to that of CAR/PAN and Zn-CAR/PAN NFs. On day 4, the cell viability of CAR/PAN NFs still significantly prevails over that of the neat PAN NFs. (b) Live/dead images and (c) DAPI/Actin staining images of the L929 cells cultured with PAN, CAR/PAN, and Zn-CAR/PAN NFs for 24 h (scale bar represents 200 and 100 μm, respectively).

The co-existence of CAR and ZnO NPs on PAN NFs was assumed to provide a proper surface chemistry and bioactivity to enable both fibroblast and endothelial cells to further proliferate. Figure 4a shows the L929 cell viability in proximity of the neat PAN, CAR/PAN, and Zn-CAR/PAN NFs. Evidently, fibroblasts are significantly (*p* < 0.05) more viable when exposed to CAR/PAN NFs on both incubation days compared to when they are treated only with PAN NFs. As mentioned earlier, CAR has been proven to provoke the healing of surgical wounds in *in vivo* studies^13^ due to its supportive role in the longevity of fibroblasts.^14^ McFarland and Holliday^53^ have validated that CAR positively affects the growth, morphology, and viability of cultured human fibroblasts (strains MRC-5 and HFF-1). Interestingly, exposure of fibroblasts to a medium with and without CAR directly changes their phenotype from senescent (w/o CAR) to juvenile (w CAR). The rejuvenation of the cells leads to the emergence of more colonies and increased growth,^53^ as clearly witnessed by us (Figures 4b and S7). As stated by Hipkiss *et al.*,^54^ the effect of CAR in raising the growth capacity of human fibroblasts originates from its potential to react with sugars. As a result, in a competitive manner, CAR hampers nonenzymic protein glycosylation. CAR has also been reported to slow replicative senescence through a delayed telomere shortening rate, thereby extending the longevity of human fibroblasts.^55^ Shao *et al.*(55) believe that CAR induces a reduction in telomere shortening rate, leading to a life-extension effect for fibroblasts. Taken together, CAR can potentially enhance the longevity and viability of fibroblasts, as widely reported in the literature. However, in our study, CAR is firmly bound to PAN NFs through hydrogen (and likely covalent) bonding and thus could act differently than free CAR does. Despite the limited exposure to the bound CAR, L929 cells still show an improved viability and colonization compared to when they are exposed to the neat PAN NFs (Figure 4b). This might arise from the improved adhesion of serum proteins, thereby enhanced cell adhesion, mediated by the presence of CAR. Furthermore, the surface charge becomes positive due to the availability of a plethora of amine functional groups of the CAR component of the NF system. The abundant presence of CAR ligands on the surface of NFs assures a positively charged surface, given that the isoelectric point of CAR is 8.2^56^ and the cell culture medium gradually turns acidic due to cell metabolism.^57^ As a result of such a charge transition, negatively charged cells are largely attracted to the CAR-loaded NFs. Figure 4c visualizes the F-actin-labeled cytoskeleton of L929 cells on the different classes of the NFs. F-actin fibers and L929 cells’ nuclei are randomly aligned, but the cytoskeleton is clearly seen on the PAN and CAR/PAN NFs. This behavior was observed in our earlier study on BSA-functionalized PAN NFs.^7^ Similarly, Kim *et al.*(58) have reported that a positively charged PCL nanomatrix supports the adhesion of NIH 3T3 fibroblast cells compared to its negatively charged counterpart. According to Keselowsky *et al.*,^59^ fibronectin, which is one of the main binding sites for the cells surface receptors (integrins), favorably adsorbs on the surfaces functionalized with NH_3_^+^ > CH_3_ > COO^–^ > OH. Therefore, carboxylated or aminated surfaces are distinct in terms of biomolecule adsorption capacity and thereby govern cell adhesion differently.

One interesting ability of CAR is its metal ion chelating activity, which was employed in our study to develop a Zn-CAR biocomplex on the surface of the hydrolyzed PAN NFs. It is well known that diabetic wound healing can be provoked by biomaterials that can chelate the Zn^2+^ ions available in the active site of matrix metalloproteinase (MMP) enzymes.^60^ The chelating ability of CAR for Zn^2+^ and Cu^2+^ has been already proven to be effective in the treatment of neuro-degenerative diseases.^61^ On the other hand, it is assumed that the biosynthesis of ZnO NPs through the chelating ability of the CAR ligands immobilized on PAN NFs can lead to the release of Zn^2+^ into the wound milieu and provide additional therapeutic effects.

As shown in Figure 4a, L929 cell viability in proximity of Zn-CAR/PAN NFs is significantly superior to that of the neat PAN NFs on day 1 (*p* < 0.05), but non-significantly lower on day 4. Compared with CAR/PAN NFs, fibroblast viability adjacent to Zn-CAR/PAN NFs is notably (*p* < 0.05) lower on both days. Such quantitative data are further validated by the live/dead stain (Figure 4b) and DAPI/Actin assay images (Figure 4c). Conclusively, both CAR/PAN and Zn-CAR/PAN NFs show improved fibroblast viability compared to the control (neat PAN NFs). While CAR can significantly raise the cell viability, ZnO NPs moderate this supportive role not through cytotoxicity (as the number of PI-labeled dead cells around Zn-CAR/PAN NFs does not change) rather *via* partially inactivating (capping) of the CAR ligands. It is also speculated that the generation of ROS by the UV-irradiated ZnO NPs (during the sterilization process) can hinder the cells’ attachment on the NFs surface and even be fatal to them. Another likely reason for the lower adhesion of L929 cells on Zn-CAR/PAN NFs could be the increased negative surface charge due to the presence of hydroxylated ZnO NPs and hydrolyzed PAN NFs (which have fewer CAR ligands and can expose their oxygen-bearing functional groups). As mentioned earlier, negatively charged cells are typically repelled from the negatively charged nanomaterials. As seen in Figure 4c, in contrast to CAR/PAN NFs, the cells are hardly able to spread on Zn-CAR/PAN NFs, most likely due to their repulsive surface charge. Figure S7 clearly shows that L929 cells adhere less to the surface of Zn-CAR/PAN NFs compared to the other NF systems. While ZnO NPs were shown to be less supportive toward the fibroblasts, they can potentially act as an antibacterial nanoelement and protect the wound against infection (as will be discussed later). Additionally, they can release Zn^2+^ ions and ROS, thereby destroying cancer cells.^62^ As Nel *et al.*(63) state, the release of Zn^2+^ ions within cells can trigger several detrimental effects, including lysosomal damage, mitochondrial perturbation, and ROS generation.

### 3.10 HUVECs Viability and Adhesion on the Bio(nano)hybrid NFs

**Figure 5:**
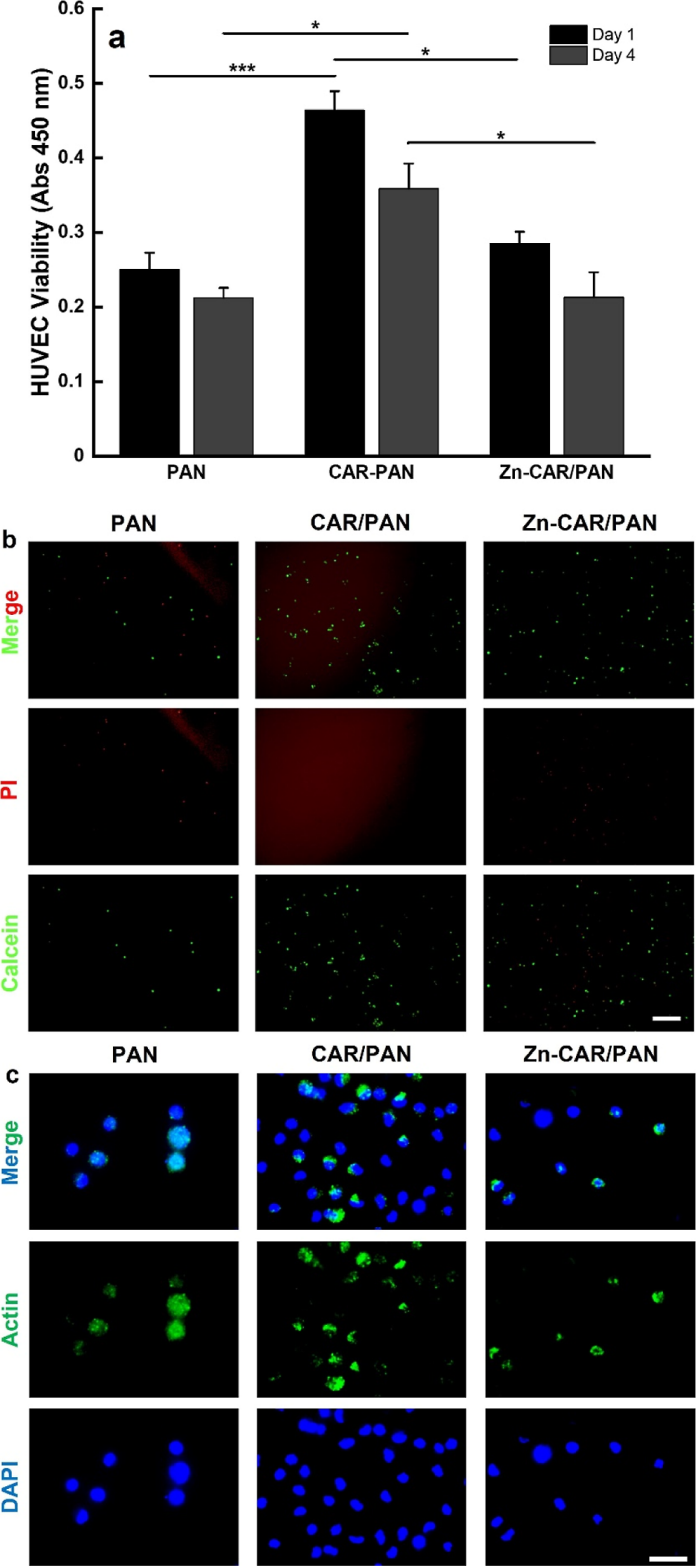
(a) HUVEC cell viability in the proximity of neat PAN, CAR/PAN, and Zn-CAR/PAN NFs (*: *p* < 0.05, ***: *p* < 0.001). (b) Live/dead images and (c) DAPI/Actin staining images of the HUVECs cultured with PAN, CAR/PAN, and Zn-CAR/PAN NFs for 24 h (scale bar represents 200 and 100 μm, respectively).

Figure 5a shows the HUVECs viability when subjected to neat PAN, CAR/PAN, and Zn-CAR/PAN NFs. Similar to the fibroblast viability, endothelial cells are significantly (*p* < 0.05 and *p* < 0.001) more viable in proximity of CAR/PAN NFs on both incubation days, compared to when treated with PAN NFs and Zn-CAR/PAN NFs. There is no significant difference in cell viability between the neat PAN and Zn-CAR/PAN NFs after 4 incubation days. However, as seen in Figure 5b, after 1 day of incubation, the population of live HUVECs cultured with Zn-CAR/PAN NFs is substantially higher than that with the neat PAN NFs. On the contrary, the number of dead cells in the presence of PAN NFs does not change compared to Zn-CAR/PAN NFs, implying that the biosynthesized ZnO NPs are non-cytotoxic. The *in vitro* cell viability test with HUVECs confirms the outcome of the fibroblast viability test and indicates that among the NF samples, the most optimum performance is achieved with CAR/PAN NFs, followed by Zn-CAR/PAN NFs. Figures 5c and S8 show the morphology of actin fibers and nuclei of HUVECs in the presence of the NF materials. Similar to fibroblasts, HUVECs’ nuclei and F-actin fibers are randomly distributed across the PAN and CAR/PAN NF mats with an almost spindle-like elongated shape. Apparently, the number of HUVECs on CAR/PAN NFs prevails over that on the neat PAN NFs. Takahashi *et al.*(15) have shown that CAR (at doses over 5 mM) can positively affect the NO production of endothelial cells, thereby promoting angiogenesis. Therefore, CAR/PAN NFs are believed to offer a promising pro-angiogenic activity. In the case of Zn-CAR/PAN NFs, HUVECs were less adhered and formed less distinct F-actin fibers on the NFs surface. Again, it turns out that the increased cell viability induced by CAR is compromised by the presence of ZnO NPs and their surface charge and, speculatively, the release of damaging ROS.

### 3.11 HUVEC Migration Behavior in the Presence of the Bio(nano)hybrid NFs

**Figure 6:**
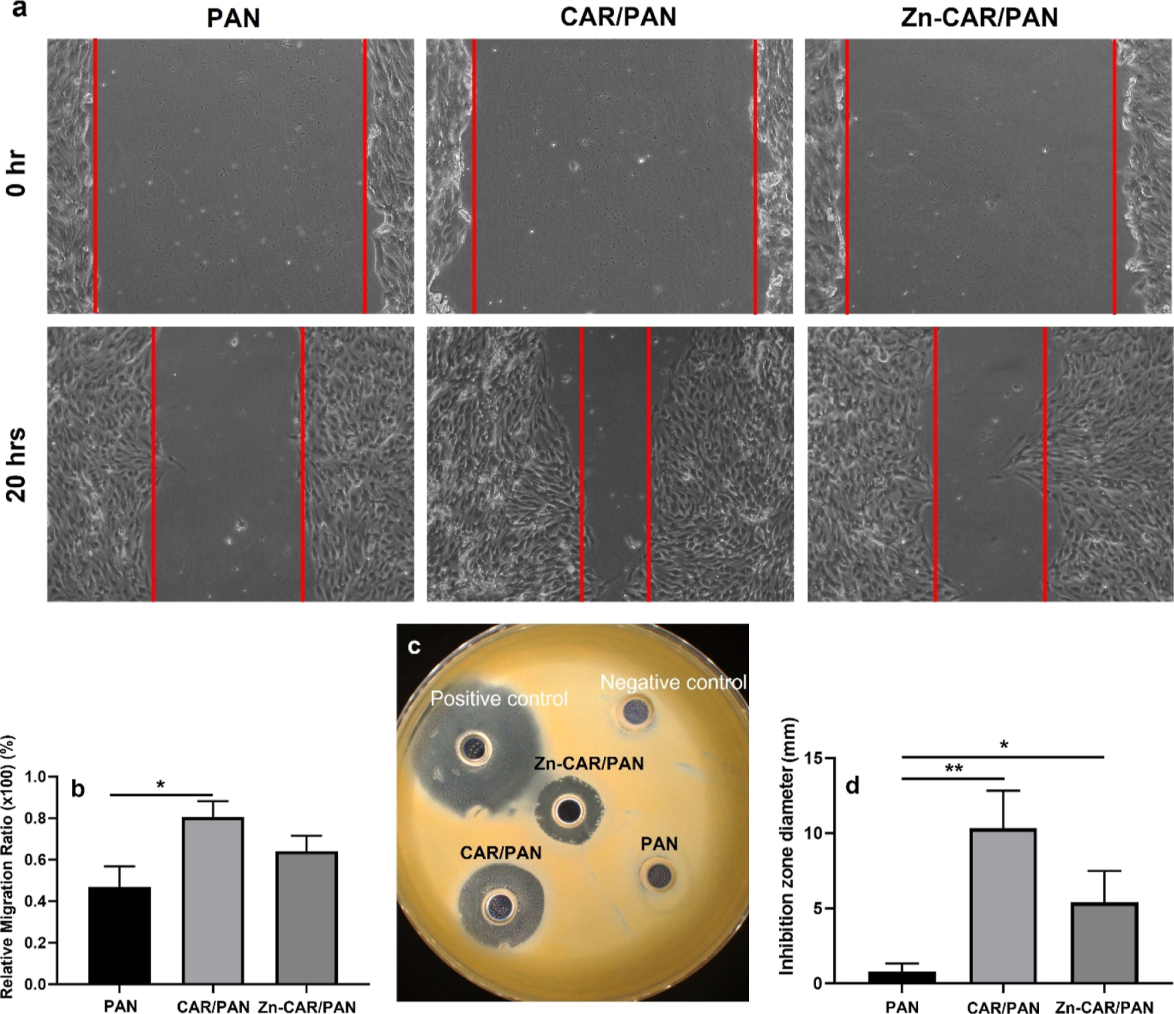
(a) Light microscopy images of the scratch area at the onset of the experiment and after 20 h. The images clearly indicate the larger migration tendency of HUVECs after incubation with CAR/PAN NFs with and without ZnO NPs compared to when they are treated with PAN NFs. (b) Relative migration ratio of HUVECs induced by PAN NFs, CAR/PAN NFs, and Zn-CAR/PAN NFs after a 20 h incubation period. The CAR/PAN NFs promote the migration extent of HUVECs significantly higher than PAN NFs do (*: *p* < 0.05). (c) Camera image shows the formation of an inhibition zone for the *S. aureus* bacteria treated with PAN NFs, CAR/PAN NFs, and Zn-CAR/PAN NFs (positive and negative controls are ampicillin and sterile water, respectively). (d) Inhibition zone diameter after exposure of *S. aureus* bacteria to PAN NFs, CAR/PAN NFs, and Zn-CAR/PAN NFs. Evidently, there is a significantly higher antibacterial activity for CAR/PAN NFs with (*: *p* < 0.05) and without (**: *p* < 0.01) ZnO NPs compared to PAN NFs.

The optimum contribution of CAR to angiogenesis is also reflected in the enhanced migration rate of endothelial cells exposed to CAR/PAN NFs. The light microscopy images of the scratch area (Figure 6a) indicate that the number of HUVECs migrating into the scratch area after 20 h incubation is greater for CAR/PAN NFs and Zn-CAR/PAN NFs compared to PAN NFs. As shown in Figure 6b, there is a significant discrepancy (*p* < 0.05) between the cell migration ratio for CAR/PAN NFs (80 ± 7%) and PAN NFs (47 ± 10%). This behavior is mainly governed by the release of CAR that encourages the proliferation and migration of endothelial cells, as discussed earlier. On the other hand, depending on concentration, the release of Zn^2+^ ions from Zn-CAR/PAN NFs can either further stimulate the migration of HUVECs or counteract CAR and partially suppress the cell migration. In this regard, Ma *et al.*(64) state that there is a Gaussian relationship between the endothelial cell migration rate and the concentration of Zn^2+^ ions in the surrounding medium. Up to 60 μM Zn^2+^ ion concentration, cell migration gradually rises and thereafter declines (particularly after 100 μM ion concentration). This ascending trend of cell migration can be related to decreased adhesion strength of HUVECs to the tissue culture plate (*i.e.*, a lower affinity between cell integrins and the available ligands on the plate surface) at low Zn^2+^ ion concentrations.^64^ The lower migration rate of the HUVECs exposed to Zn-CAR/PAN NFs compared to CAR/PAN NFs might be associated with the high concentration of Zn^2+^ ions that potentially strengthen the cell adhesion to the culture plate surface.

### 3.12 Antibacterial Potential of the Bio(nano)hybrid NFs

As a multifunctional NF wound dressing, it would be optimal if the CAR/PAN NFs with and without ZnO NPs not only support cellular activities but also reduce the bacteria commonly found in the wound milieu. Alongside *Pseudomonas aeruginosa*, *S. aureus* is the most widely available bacteria in chronic wounds that can release virulence factors and surface proteins, thereby challenging wound healing.^65^Figure 6c shows a camera image of the bacterial inhibition zones caused by the leach liquor of PAN NFs, CAR/PAN NFs, and Zn-CAR/PAN NFs. Visually, the inhibition zone of CAR/PAN is the largest zone, followed by that of Zn-CAR/PAN NFs. The inhibition zone of CAR/PAN NFs with (6 ± 2 mm) and without (11 ± 3 mm) ZnO NPs is significantly larger than that of PAN NFs (1 ± 0.1 mm) (*p* < 0.05 and *p* < 0.01, respectively) (Figure 6d). Figure S9 shows the merged live/dead staining images of the *S. aureus* bacteria treated with PAN, CAR/PAN, and Zn-CAR/PAN NFs’ liquid extracts for 24 h. The images further verify the highest effectiveness of CAR/PAN NFs in inactivating the bacteria among the three NF groups. Evidently, Zn-CAR/PAN NFs are superior to PAN NFs in terms of antibacterial activity, as reflected in the much larger number of dead bacteria. The antibacterial activity of CAR as a biofunctional agent coupled with other nanomaterials such as GO has been already reported.^66^ It has been postulated that the antibacterial effect of CAR originates from its histidine subunit, which is a cationic amino acid with an imidazole ring and can inhibit bacterial adhesion and thus biofilm formation. In addition, CAR can inactivate bacteria by hampering their glucosyltransferase activity.^66^

The antibacterial potential of ZnO NPs is well-established, and these NPs have been proven to be more effective compared to other metal oxide NPs, including MgO, TiO_2_, Al_2_O_3_, CuO, and CeO_2_.^67,68^ The antibacterial activity of ZnO NPs is notably size-dependent. For instance, as reported by Jones *et al.*,^68^ small ZnO NPs (8 nm in diameter) show over 95% bacterial (*S. aureus*) growth inhibition at 1 mM concentration, while 5 mM of larger ZnO NPs (50–70 nm in diameter) could induce 40–50% bacterial growth inhibition. The small ZnO NPs, as is the case in our study, lead to disintegration of bacteria’s membrane, thus allowing for large cellular internalization, and release a high density of ROS (OH radicals) in the surrounding aqueous medium, thereby inactivating bacteria.^67^

Despite the promising antibacterial potential of CAR and ZnO NPs individually, the co-existence of these components does not bring about an improved antibacterial efficiency compared to CAR alone. As reported by Applerot *et al.*,^67^ at a minor concentration of ZnO, bacteria can partially solubilize ZnO and metabolize the bioavailable Zn^2+^ ions as an oligoelement.^69^ Zn^2+^ homeostasis is vital for bacterial life, as Zn^2+^ ions are involved in the regulation of a variety of metabolic activities as catalysts, cofactors, and coenzymes. Moreover, Zn^2+^ ions stabilize DNA-binding proteins and enzymes.^70^ On the other hand, excess Zn^2+^ ions are fatal for bacteria. Therefore, various bacteria, including *S. aureus,* are so evolved to adjust the influx/efflux processes, thereby sustaining a desirable intracellular concentration of Zn^2+^ ions.^68^ Additionally, CAR, as an antioxidant, can neutralize the ROS generated by ZnO NPs. Inactivation of some part of CAR by ROS can result in less antibacterial activity compared to CAR alone.

## 4 Conclusions

For the first time, CAR was immobilized on hydrolyzed PAN NFs to develop a biohybrid NF system with a promising wound healing potential reflected in the improved viability of fibroblasts and endothelial cells. Additionally, CAR enabled the efficient biosynthesis of ultrafine ZnO NPs that were uniformly distributed across the NF mat. ZnO NPs were meant to release the Zn^2+^ ion, which is an essential trace element involved in the chemistry of different transcription factors or enzymes that contribute to cell proliferation. However, the presence of ZnO NPs did not promote the cell viability compared to the biohybrid NFs but rather reduced viability. Yet, the live/dead imaging of the cells cultured with Zn-CAR/PAN NFs implied a negligible (or even no) cytotoxicity for the CAR/PAN NFs loaded with ZnO NPs. This finding contradicts the outcome of many *in vitro* cytotoxicity tests that have confirmed the adverse consequences of ZnO NPs on cells.^62^ In addition to a wound healing effect, thanks to the small size of ZnO NPs and the possibility of the generation of ROS and the release of Zn^2+^ ions and CAR into the medium, the bionanohybrid NFs could also perform as an antibacterial wound dressing material. It is worth noting that, to the best of our knowledge, no earlier study has investigated the antibacterial effect of CAR coupled with ZnO NPs. Benefitting from the photocatalytic activity of ZnO NPs when UV-irradiated, it is speculated that this biomimetic NF wound dressing can not only promote skin regeneration (thanks to CAR) but also inhibit tumor growth, thereby enabling an effective post-surgical treatment for melanoma. The anticancer properties of Zn-CAR/PAN NFs are being investigated currently, and results will be disseminated promptly.

## Supporting Information Available

Camera image of the synthesized Zn-CAR/PAN NF mat; NF diameter histogram for neat PAN NFs, CAR/PAN NFs, and Zn-CAR/PAN NFs; the surface morphology of the unwashed Zn-CAR/PAN NFs at two different magnifications; EDX general elemental map ; EDX data verify the presence of Cl alongside Zn and O, implying the formation of a coating material composed of ZnCl_2_ and ZnO together; porosity measured *via* the ImageJ software for neat PAN NFs (17%), CAR/PAN NFs (6%), and Zn-CAR/PAN NFs (8%); water contact angle measurement on neat PAN NF mat, hydrolyzed PAN NF mat, CAR/PAN NF mat, and Zn-CAR/PAN NF mat; water droplet has been instantly absorbed into the NF mats; stress–strain curves of PAN, c-PAN, CAR/PAN, and Zn-CAR/PAN NFs; DAPI/Actin staining of the L929 cells co-cultured with the PAN, CAR/PAN, and Zn-CAR/PAN NFs for 24 h; DAPI/Actin staining of the HUVECs co-cultured with the PAN, CAR/PAN, and Zn-CAR/PAN NFs for 24 h (PDF)
