# Supplementary material for: Biosynthesis of Zinc Oxide Nanoparticles on l-Carnosine Biofunctionalized Polyacrylonitrile Nanofibers; a Biomimetic Wound Healing Material
Source: ACS Appl Bio Mater. 2023 Sep 18;6(10):4290–303. doi: 10.1021/acsabm.3c00499 (PMC10583230; doi:10.1021/acsabm.3c00499)
Supplement: Supplementary file 1 — mt3c00499_si_001.pdf [file mt3c00499_si_001.pdf]

Supplementary Information

Biosynthesis of zinc oxide nanoparticles on L-carnosine biofunctionalized polyacrylonitrile nanofibers; a biomimetic wound healing material

Shahin Homaeigohar [a, *], Mhd Adel Assad [b], Amir Hossein Azari [b], Farnaz Ghorbani [c], Chloe Rodgers [d], Matthew J Dalby [d], Kai Zheng [e], Rongyao Xu [e, f, *], Mady Elbahri [b, *], Aldo. R. Boccaccini [c]

a: School of Science and Engineering, University of Dundee, Dundee DD1 4HN, United Kingdom.
b: Nanochemistry and Nanoengineering, Department of Chemistry and Materials Science, School of Chemical Engineering, Aalto University, 02150 Espoo, Finland.
c: Institute of Biomaterials, Department of Materials Science and Engineering, University of Erlangen-Nuremberg, 91058 Erlangen, Germany.
d: Centre for the Cellular Microenvironment, University of Glasgow, Glasgow, 11 6EW, United Kingdom.
e: Jiangsu Province Engineering Research Center of Stomatological Translational Medicine, Nanjing Medical University, Nanjing 210029, China.
f: Department of Oral and Maxillofacial Surgery, Stomatological Hospital, Nanjing Medical University, Nanjing 210029, China.

Corresponding authors: Shahin Homaeigohar (Shomaeigohar001@dundee.ac.uk), Rongyao Xu (rongyaoxu@njmu.edu.cn), and Mady Elbahri (mady.elbahri@aalto.fi).

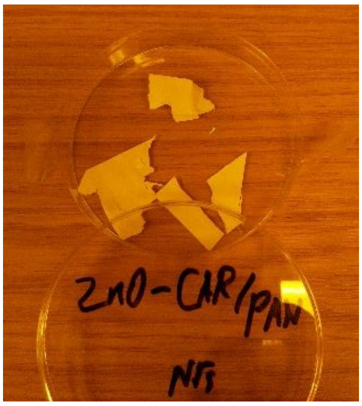

Figure S1. A camera image of the synthesized Zn-CAR/PAN NF mat.

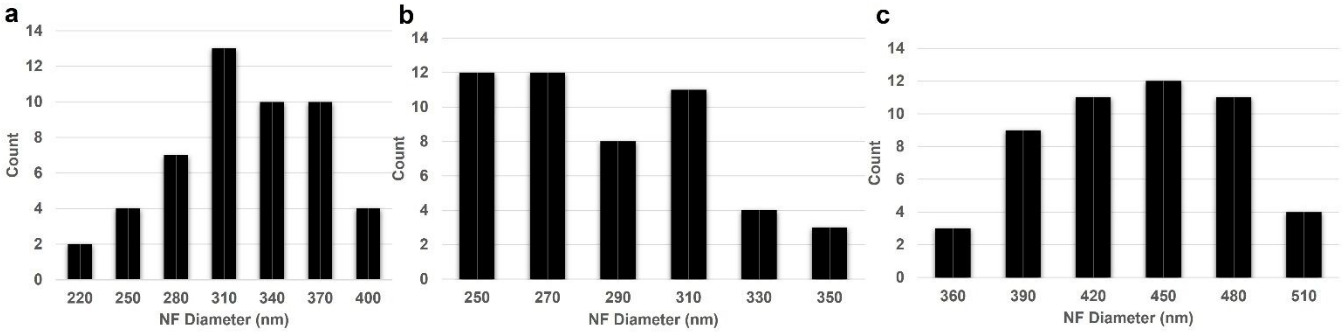

Figure S2. NF diameter histogram for: a) neat PAN NFs, b) CAR/PAN NFs, c) Zn-CAR/PAN NFs.

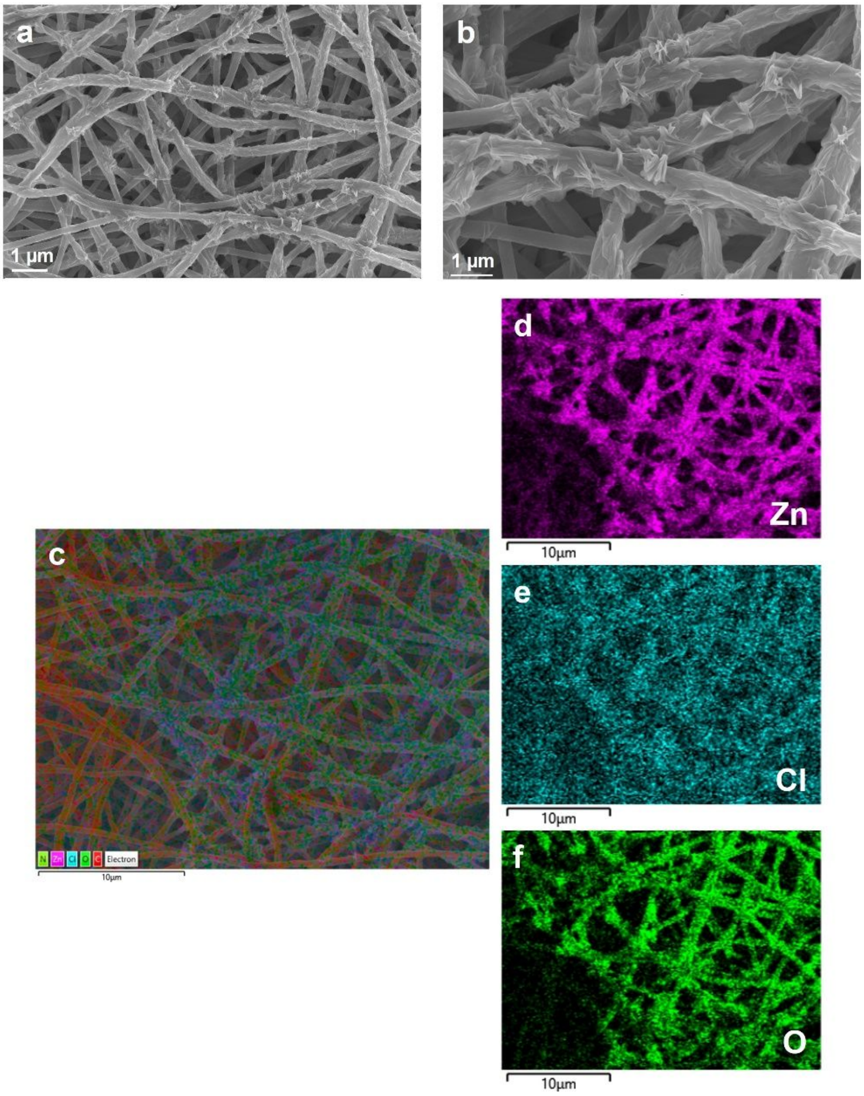

Figure S3. The surface morphology of the unwashed Zn-CAR/PAN NFs at two different magnifications (a &b). c) EDX general elemental map (the elements have been represented by colourful dots. N might indicate the amine groups of CAR), d) corresponding Zn map of (c), e) corresponding Cl map of (c), and f) corresponding O map of (c). The EDX data verify the presence of Cl alongside Zn and O, implying the formation of a coating material composed of $ZnCl_2$ and ZnO together.

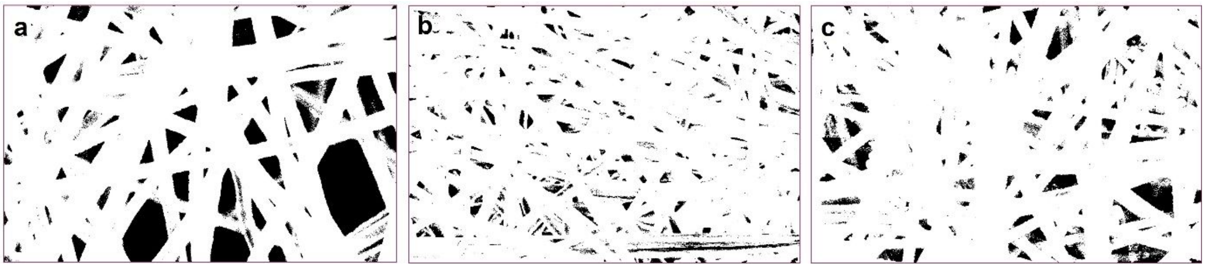

Figure S4. Porosity measured via the ImageJ software for: a) neat PAN NFs (17%), b) CAR/PAN NFs (6%), c) Zn-CAR/PAN NFs (8%).

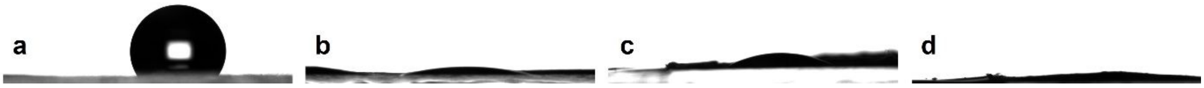

Figure S5. Water contact angle measurement on: a) neat PAN NF mat, b) hydrolysed PAN NF mat, c) CAR/PAN NF mat, and d) Zn-CAR/PAN NF mat. b-d) water droplet has been instantly absorbed into the NF mats.

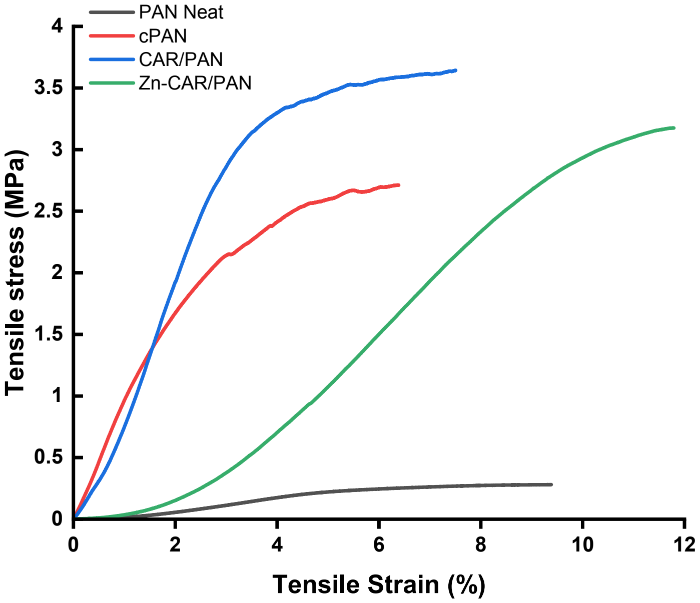

Figure S6. Stress-strain curves of PAN, c-PAN, CAR/PAN, and Zn-CAR/PAN NFs.

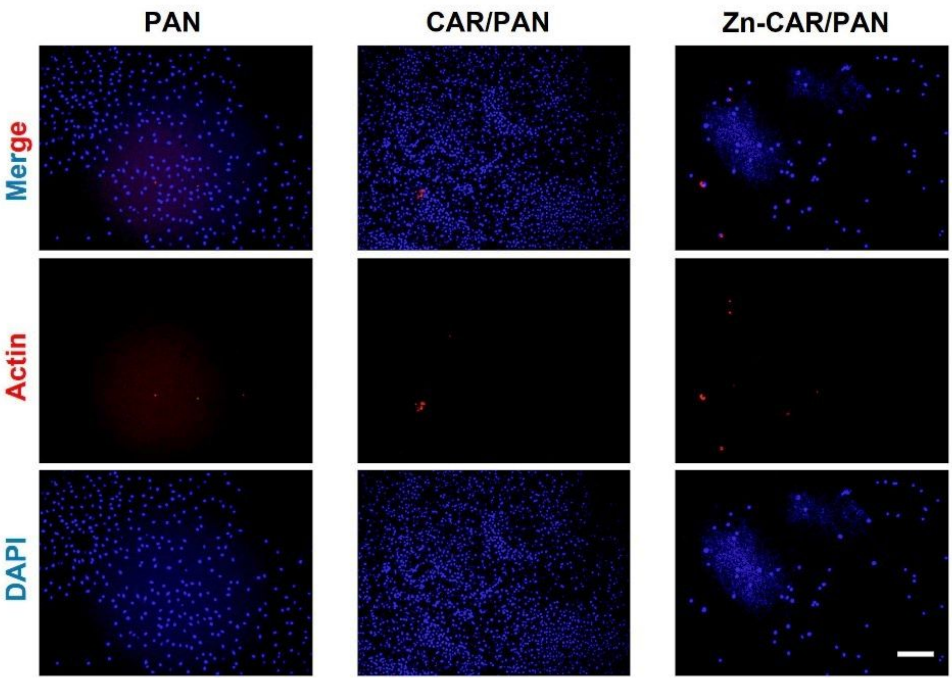

Figure S7. DAPI/Actin staining of the L929 cells cultured with the PAN, CAR/PAN, and Zn-CAR/PAN NFs for 24 hours (scale bar represents 200 μm).

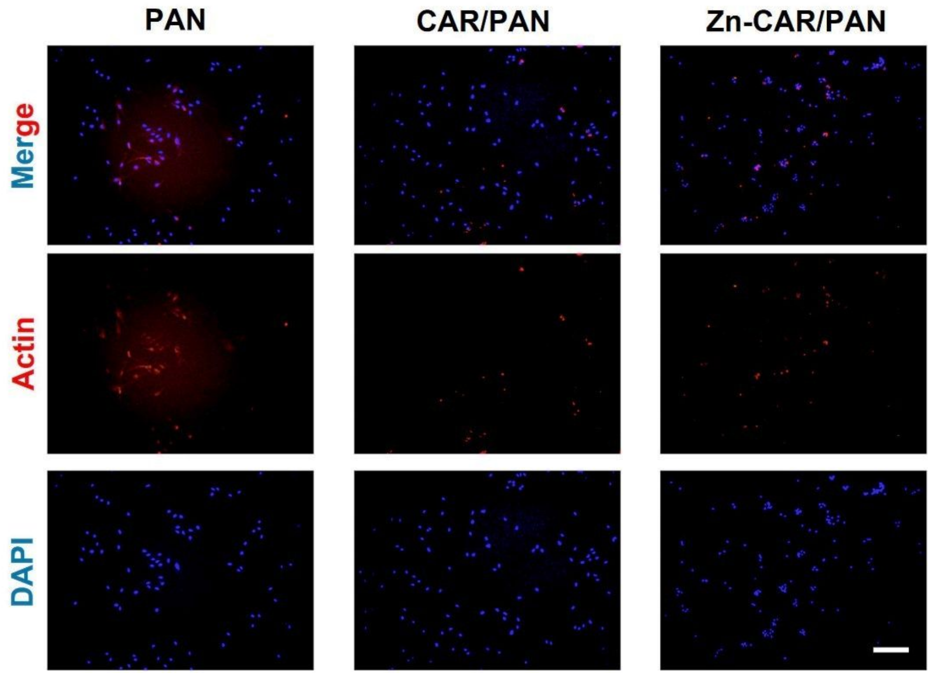

Figure S8. DAPI/Actin staining of the HUVECs cultured with the PAN, CAR/PAN, and Zn-CAR/PAN NFs for 24 hours (scale bar represents 200 μm).

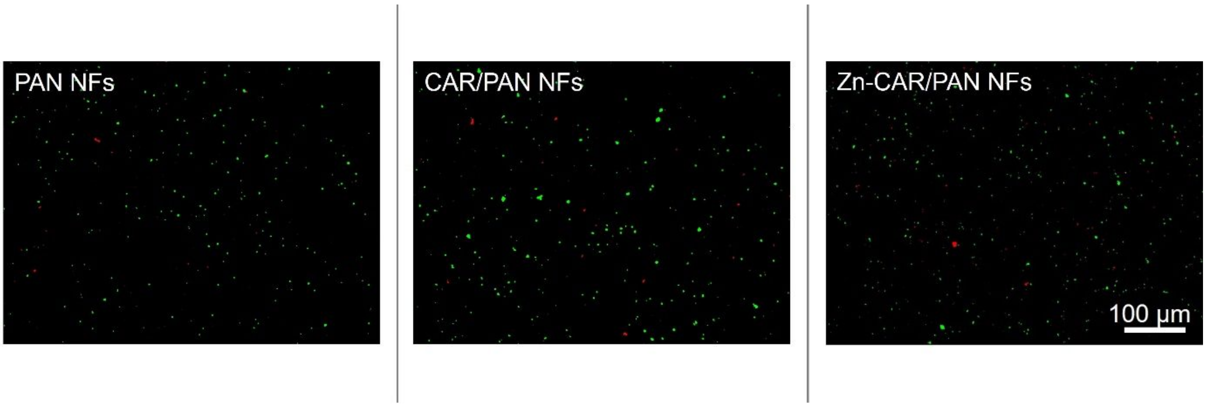

Figure S9. Merged Live/Dead staining images of the *S. aureus* bacteria treated with the liquid extract of PAN, CAR/PAN, and Zn-CAR/PAN NFs for 24 hours.
